# High repetitive arginine in the anterior of PCV3 capsid protein is a severe obstacle for its expression in *E. coli*

**DOI:** 10.1186/s13568-020-01163-8

**Published:** 2020-12-11

**Authors:** Bing Yan Liu, Bin Gao, Meng Zhi Liu, Ting Ting Zhang, Bao Shan Liu, Ze Liang Chen

**Affiliations:** Key Laboratory of Livestock Infectious Diseases in Northeast China, Ministry of Education, College of Animal Science and Veterinary Medicine, Shenyang Agricultural University, No. 120, Dongling Road, Shenyang, 110866 Liaoning People’s Republic of China

**Keywords:** PCV3, Capsid protein, Expression, High repetitive, Arginine, Obstacle

## Abstract

Porcine circovirus type 3 (PCV3) is a novel circovirus identified in sows with PDNS-like clinical signs and reproductive failure. The capsid protein (CAP) of PCV3 is expected to be an effective vaccine candidate. Here, we expressed the original capsid protein, truncated capsid protein without anterior highly repetitive arginine (ΔCAP) and their codon-optimized counterparts in *E. coli*. These results showed that lots of repeated arginine could severely lower the expression of PCV3 capsid protein in *E. coli*. At the same time, the recombined truncated PCV3 capsid protein forms typic virions. The efficient expression of capsid protein is expected to serve the development of PCV3 vaccines and other studies of PCV3 capsid protein.

## Key points

1. High repetitive arginine in the anterior of PCV3 CAP severely hinders its expression in *E. coli*.
2. The purified recombined ΔCAP automatically assemble into typic virions.
3. The efficient expression of CAP serves studies of PCV3.

## Introduction

Circoviruses are a class of the smallest DNA viruses, with a particle size of about 20 nm. Porcine circoviruses include Porcine circovirus 1 (PCV1), PCV2: Porcine circovirus 2 (PCV2), and Porcine circovirus 3 (PCV3). PCV3 is a novel circovirus identified in 2017 in sows with reproductive failure and clinical signs like pig dermatitis and nephrotic syndrome (PDNS) (Palinski et al. 2017). The capsid and replicase proteins of PCV3 are only 37% and 55% identical to PCV2 and bat circoviruses, respectively (Palinski et al. 2017). After the first confirmed case in the USA in 2017, PCV3 was subsequently identified in pigs in other countries such as China (Zhai et al. 2017), Italy (Faccini et al. 2017), Poland (Stadejek et al. 2017), Korea (Kwon et al. 2017), Russia (Yuzhakov et al. 2018), Japan (Hayashi et al. 2018), Brazil (Tochetto et al. 2018) Denmark Spain (Franzo et al. 2018) and Sweden (Ye et al. 2018). PCV3 DNA was even detected in the dog (4 of 44 dogs) (Zhang et al. 2018). It is also found that the PCV3 infection increased rapidly from late 2013 to early 2014 (Li et al. 2018). The high occurrence of PCV3 may pose a potential threat to the swine industry worldwide (Li et al. 2018). However, the processes resulting in the emergence and spread of PCV3 remain poorly understood (Li et al. 2018). Until today PCV3 has not been successfully cultured in vitro (Deng et al. 2018), which makes it difficult to study its pathogenesis, protein function, and vaccine.

To further study the structure and function of the virus and to develop a vaccine, the viral coat protein needs to be expressed. Prokaryotic expression in *E. coli* is the most mature and efficient expression method. However, in our previous study, the PCV3 capsid protein was barely expressed in *E. coli*. So to understand the influence factor for the expression of the PCV3 capsid protein, the expression of PCV3 capsid protein in *E. coli* was tested after codon optimization and truncation.

## Methods

### Analysis of the sequence of the capsid protein

For high levels of expression in *E. coli*, the codon usage of the gene sequence of the capsid protein of PCV3 isolate (MK000387) was analyzed at the online *E. coli* Codon Usage Analysis 2.0 (http://faculty.ucr.edu/~mmaduro/codonusage/usage.htm.). Then antigenic epitopes of the PCV3 capsid protein (CAP) were analyzed by the online program Bepipred Linear Epitope Prediction 2.0 and ElliPro at the website immune Epitope database and analysis resource (IEDB) (http://www.iedb.org/). Based on the analysis of the sequence, the intact PCV3 CAP and a truncated Capsid protein without anterior 32 amino acids (ΔCAP) were selected to express. At the same time, both amino acid sequences were codon-optimized for expression in *E. coli* utilizing the online Reverse Translate program of the Sequence Manipulation Suite (http://www.bioinformatics.org/sms2).

### Construction of expression vector of the capsid protein

The codon optimization sequences of CAP and ΔCAP were synthesized and inserted into *Bam*H I and *Xho* I site of a pET-28a plasmid vector respectively to construct the recombined plasmid pET-3CAP-O and pET-Δ3CAP-O by Sangon Biotech (Shanghai) Co., Ltd.

Using DNA samples identified as PCV3 positive by PCR (Palinski et al. 2017) as templates, the gene sequences of CAP and ΔCAP were amplified by Polymerase chain reaction (PCR) with two pairs of primer (Table 1). After digested by the restriction enzyme *Bam*H I and *Xho* I, they were cloned into pET-28a plasmid vector respectively to construct the recombined plasmid pET-3CAP and pET-Δ3CAP. The recombinant plasmid was introduced into *E. coli* BL21 (DE3) cells by transformation using traditional chemical transformation methods.

**Table 1:** The primers for the sequences of CAP and ΔCAP

Probe/primer	The DNA sequence (5′-3′)	Tm	Amplicon size (bp)
CAPF	tttt ggatcc ATGAGACACAGAGCTATATTCAG	55.40	650
ΔCAPF	tttt ggatcc CCCACAGCTGGCACATAC	58.09	554
CAPR	tttt ctcgag TTCACTTAGAGAACGGACTTG	55.31	

The lowercase letters are protective bases and restriction sites, respectively

The recombined bacteria with the plasmid pET-28a, pET-3CAP, pET-Δ3CAP, pET-3CAP-O and pET-Δ3CAP-O were cultured respectively in 5 mL ZYP-5052 medium in 37 ℃ until the OD_600_ of the cultures reached 0.6. Then they were transferred to 20 °C for 12 h to induce the expression of the recombined protein (Studier 2005).

### Expression of the recombined capsid and truncated capsid protein

**Fig. 1:**
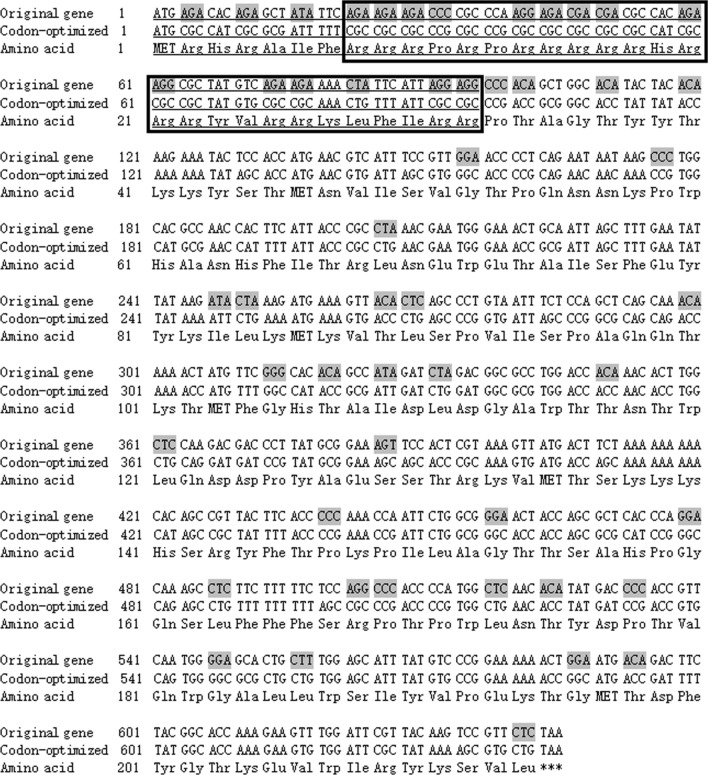
Expression and identification of recombinant PCV3 capsid proteins expressed in *E. coli*. **a** The SDS-PAGE analysis of the recombined capsid protein of PCV3. M, protein marker; 1, control bacteria with pET-28a; 2, the bacteria with pET-3CAP; 3, the bacteria with pET-Δ3CAP; 4, the bacteria with pET-3CAP-O; 5, the bacteria with pET-Δ3CAP-O. **b** Western blots analysis of the expressed recombined proteins using the anti-His antibody. The blot corresponds to lanes 1–5 from (**a**). The arrows represent the location of the recombined ΔCAP (25 kDa), respectively. The expression of the recombined CAP protein did not be detected

The expressed cells were harvested by centrifugation and mixed with sodium dodecyl sulfonate-polyacrylamide gel electrophoresis (SDS-PAGE) loading buffer containing β-mercaptoethanol for the SDS-PAGE, then loaded on 12% SDS-PAGE after heating the samples for 10 min at 100 °C. The polyacrylamide gel was stained using Coomassie Blue for visualizing the protein. The result was taken a photo (Fig. 1a) on a Tanon GIS system (Tanon, China), and protein expression was analyzed quantitatively by the ImageJ software (NIH, USA) basing on the proportion of their grey value in the total grey value.

The recombinant proteins resolved on SDS-PAGE gels were also transferred to the nitrocellulose membrane (Whatman, Germany) using the Mini Trans-Blot Transfer Unit (BIO-RAD, USA) for one hour. The membrane was blocked with 1% bull serum albumin (BSA) for 1 h at room temperature and incubated at room temperature for half an hour with anti-his tag monoclonal antibody (Sangon Biotech Co., Ltd., Shanghai, China) (1:5000) in 1% BSA. After being washed with PBST (50 mM potassium phosphate, 150 mM NaCl, 0.05% Tween 20, pH 7.2) for three times at 5 min interval, the membrane was incubated with a 1:2000 horseradish peroxidase (HRP)-conjugated goat anti-mouse IgG for 0.5 h at room temperature. After further washing, the signal was detected using the colourimetric substrate DAB/H_2_O_2_ in the dark place for 15 min.

### Purification and identification of the recombinant PCV3 capsid protein

Wet induced cells of the recombinant *E. coli* expressing ΔCAP were harvested by centrifugation at 5000×*g* for 10 min. The bacterial pellet was washed and resuspended in binding buffer (20 mM sodium phosphate, 500 mM NaCl, 20–40 mM imidazole, pH 7.4). Cell paste was lysed over 15 passages through the cell disrupter JN-Minipro (JUNENG NANO&BIO TECH.CO., LTD, Guangzhou, China) at 1000 bar pressure. Cell-lysate was clarified by centrifuging at 12,000×*g* for 10 min to remove the cell debris. Then the supernatant was loaded to a HisTrap FF column (GE, USA), which was connected to an AKTA prime chromatography system (GE Healthcare, Chalfont St. Giles, United Kingdom). After washing with 10 column volumes of binding buffer, the ΔCAP protein was eluted with the binding buffer containing 50, 100, 300 mM imidazole, pH 7.2. The collected fractions were identified by SDS-PAGE gels and Western-blot assay with positive serum of PCV3 and HRP-conjugated goat anti-pig IgG (No.D111051, Sangon Biotech Co., Ltd., Shanghai, China).

### Transmission electron microscopy (TEM)

Two µg of purified ΔCAP were adsorbed onto a carbon-coated copper grid and incubated for about 1 min. Then, grids were dried using filter paper, negatively stained with 2% of phosphotungstic acid (PTA) for about 40 s, and viewed using an HT7700 transmission electron microscope (Hitachi, Japan), operating at 120 kV.

## Results

### Analysis of the sequence of the capsid protein

**Fig. 2:**
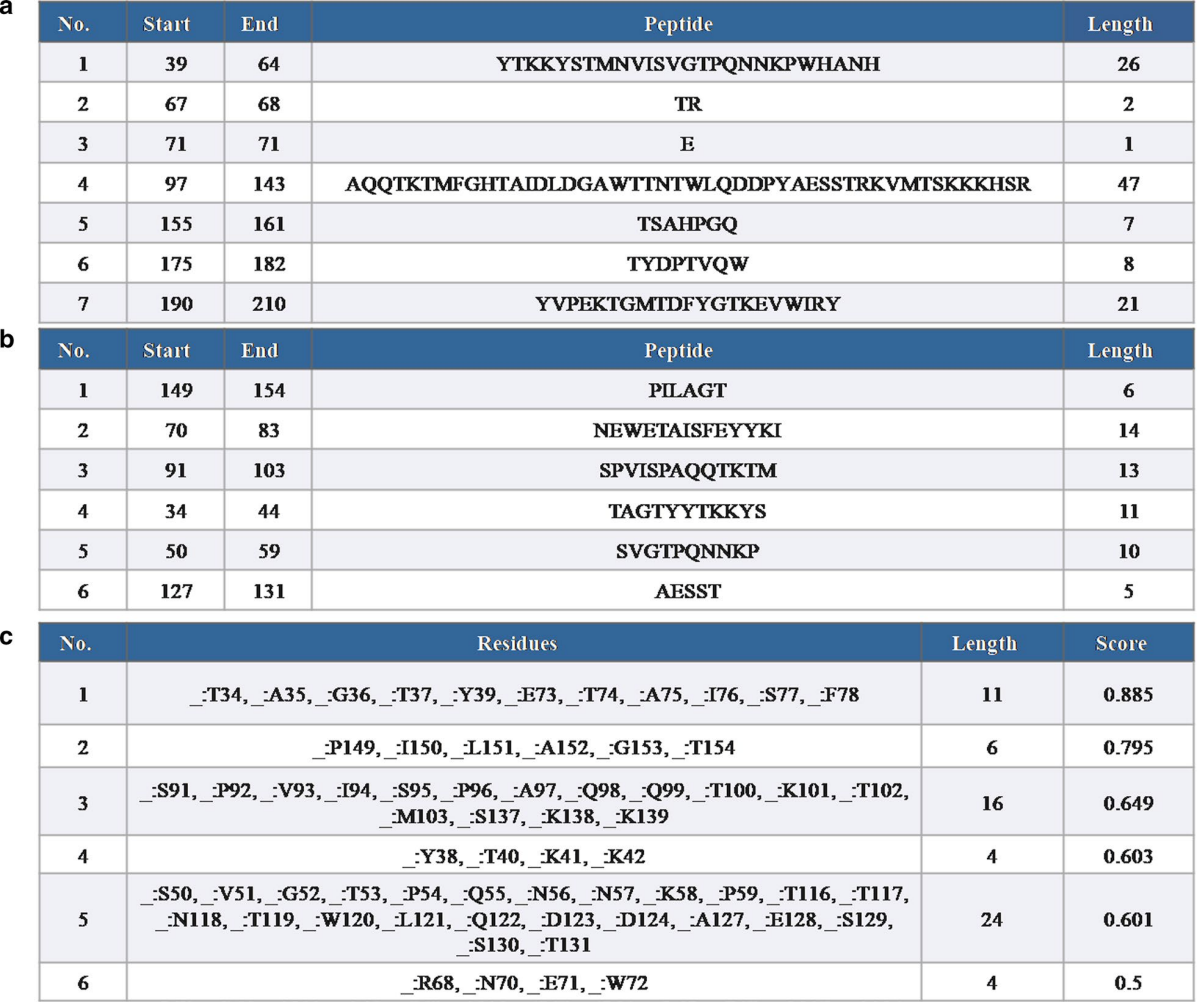
Codon analysis of PCV3 capsid protein. The original gene of CAP protein has 23% rare codon (51/214) analyzed by online *E. coli* Codon Usage Analysis 2.0, which were replaced in the codon-optimized sequence without changing the amino acid sequence. The codon in the shadow represents a rare codon. The underlined sequence was removed in the truncated CAP protein (ΔCAP). The concentrated arginines area is in the box

The analysis of the codon usage of the sequence of PCV3 capsid protein showed that 23% (51/214) of the PCV3 codons were rare codons for *E. coli* (Fig. 2). The N-terminal of the capsid protein is especially rich in arginine, where there are 22 arginines among the top 40 amino acids, accounting for 55%.

**Fig. 3:**
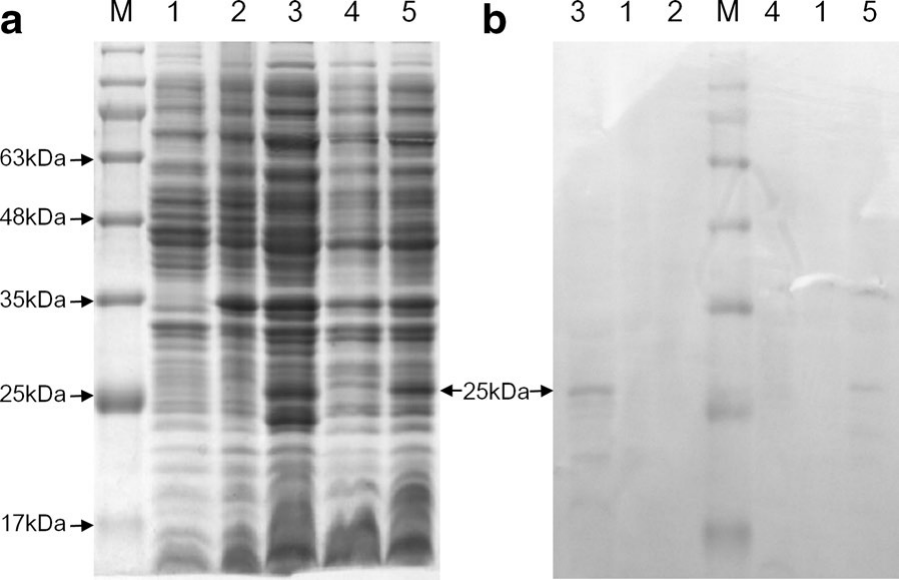
Antigen Epitope analysis of PCV3 capsid protein at the website immune Epitope database and analysis resource (IEDB). **a** A list of linear epitopes predicted by Bepipred Linear Epitope Prediction 2.0; **b** A list of linear epitopes predicted by ElliPro; **c** A list of discontinuous B cell epitopes predicted by ElliPro

The results of the antigenic epitopes predicted by the online program Bepipred Linear Epitope Prediction 2.0 and ElliPro found that no linear epitopes or discontinuous B cell epitopes were detected in the first 33 amino acids of the coat protein, which indicates that the N terminal 33 amino acids can be deleted without affecting antigenicity of the protein (Fig. 3). So, to determine the influence of high repetitive arginine in the expression of the *E. coli*, the intact PCV3 Cap (CAP) and a truncated Cap without anterior 32 amino acids (ΔCAP) were selected to express. At the same time, the codon-optimized Cap gene sequence was submitted into the GeneBank as Gene Accession Number MT452640. The codon-optimized ΔCAP gene sequence came from the codon-optimized Cap gene. They all were expressed to determine the influence of rare codon and dense arginine by comparison with that of the innate CAP and ΔCAP gene.

### Expression of recombining capsid protein

The construct recombined bacteria containing the pET-28a, pET-3CAP-O, pET-Δ3CAP-O, pET-3CAP, and pET-Δ3CAP vector were cultured in 5 mL ZYP-5052 medium for 24 h for expression of capsid protein. Then 1 g expressed cells harvested were used for the SDS-PAGE. The results showed that two obvious 25 kD protein bands appeared in the lands of recombined bacteria pET-Δ3CAP-O and pET-Δ3CAP, respectively, whereas the lands of negative bacteria and recombined bacteria pET-28a, pET-3CAP-O and pET-3CAP had no corresponding bands (Fig. 1a). It indicated that the codon optimation of CAP did not increase its expression in *E. coli*, but the truncation did. The grey value analysis of bands in the photo of SDS-PAGE showed that the recombinant ΔCAP in the bacteria containing pET-Δ3CAP-O and pET-Δ3CAP were constituted 6.2% and 6.1% of the total bacteria proteins, respectively (Table 2). It showed that the optimization of codon usage increased the expression of capsid protein to a negligible extent. On the other hand, it further reflects the extent to which the truncated processing improves the expression. In the western-blot, the proteins in both bands of ΔCAP were recognized by the antibody against 6 His-tag, respectively, but no band appears in both lanes of recombined CAP (Fig. 1b). It further showed that the optimization of codon did not affect the expression.

**Table 2:** The grey value of expressed ΔCAP band and total bacteria analyzed by the ImageJ software

	Bacteria with plasmid pET-Δ3CAP	Bacteria with plasmid pET-Δ3CAP-O
Expressed ΔCAP	8067.335	5409.92
Total	132229.8	87124.77
Percent of expressed ΔCAP	6.1%	6.2%

### Purification of the recombinant ΔCAP protein

**Fig. 4:**
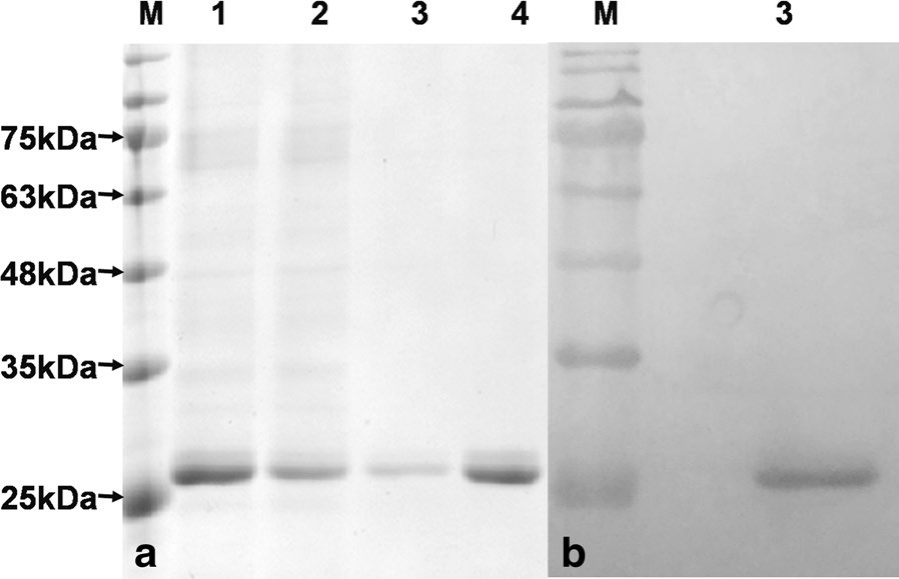
Analysis of the purified ΔCAP.A, The ΔCAP-collection profile of HisTrap FF column. 1, Lysis sample before purification; 2, the sample passed through the column; 3–4, Elution sample with eluate containing 100 and 300 mM imidazole, respectively; M: protein marker. B, Western blot analysis of purified protein using positive swine sera of PCV3 infection

The recombinant ΔCAP from *E. coli* with the pET-Δ3CAP-O plasmid was purified using Ni–NTA column according to the manufacturer’s instruction. SDS-PAGE gels identified three fractions. The ΔCAP with a purity of 95% mainly appeared in the elution with 100 mM imidazole and migrated as one defined band with 26 kDa (Fig. 4a). Western-Blot assay with positive serum of PCV3 and goat anti-pig IgG (Sangon Biotech Co., Ltd., Shanghai, China) showed that the positive serum of pig also recognized the proteins in the bands with PCV3 infection (Fig. 4b). It manifested that the recombine protein is the capsid protein of PCV3, and it has immunoactivity.

### Transmission electron microscopy (TEM)

**Fig. 5:**
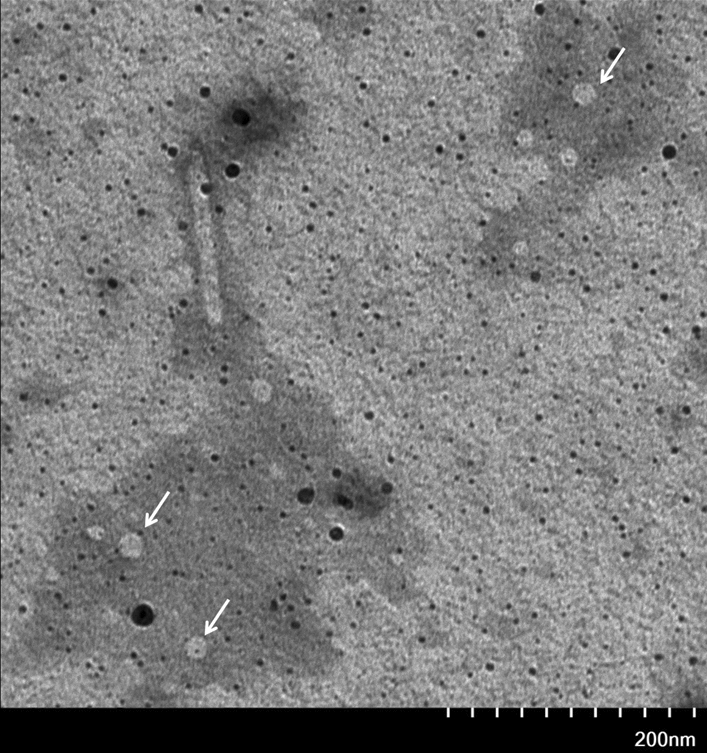
Transmission electron micrographs of ΔCAP. Virus-like particles (VLPs) formed by purified ΔCAP proteins expressed in *E. coli* cells were viewed using an HT7700 operating at 120 kV

The purified ΔCAP in the elution with 100 mM imidazole was observed under transmission electron microscopy (TEM). There existed numerous particles with similar morphology of circovirus (Xiao et al. 2018) and the diameter ranging from 15 to 20 nm (mean 17 nm), which were the same homogenous in size and morphology as PCV2 virus particles (Fig. 5). So, the result showed that the ΔCAP purified from the *E. coli* cells was assembled into virus-like particles (VLP).

## Discussion

In our present study, we investigated which factor of the rare codon or the dense arginine is a more important obstacle in the expression of PCV3 Cap protein. To clarify it, innate, codon-optimized, truncated, truncated and codon-optimized CAP proteins were expressed in *E. coli*. Our results showed that the deletion of anterior high repetitive arginine could improve the expression of Cap protein in *E. coli* and didn’t influence the formation of virus-like particles.

Rare codon analysis showed that the anterior N-terminal of PCV3 capsid protein contained a large number of rare codons, which were concentrated and repeated (Fig. 2). For the expression of a protein in the pET28a expression vector, the number of rare codons and their concentration degree are the important affecting factors (Karimi et al. 2015; Nouri et al. 2016). So the innate Cap protein almost wasn’t expressed in *E. coli*, while the truncated Cap protein was obviously expressed.

Moreover, after optimizing the rare codon, the expression of the whole and truncated Cap still showed a huge difference. Although the truncated CAP protein still contained a large number of rare codons, its expression was similar to the codon-optimized truncated cap protein. This demonstrated that highly repetitive arginine is a more obvious obstacle in the expression of PCV3 capsid protein in *E. coli* than rare codons. To the best of my knowledge, this is the first report of the effects of repeat codons in the expression of PCV3 Cap protein. Whether this effect also occurs on other amino acids in other proteins requires further study.

It is reported that PCV2 capsid protein without a nuclear localization signal (NLS) still forms virus-like particles (VLP), which were less homogenous in size and morphology than the typic circovirus (Zhou et al. 2005; Xiao et al. 2018). The VLP formation of purified PCV3 ΔCAP was also examined in this study. The VLPs of ΔCAP had typic circovirus morphology and size. The differences of VLP of truncated Cap protein of PCV2 and PCV3 may be due to viral proteins and truncated region.

PCV3 is being a new focus of attention in all pig-producing areas of the world, so developing a vaccine is a need. Because PCV3 has not been successfully cultured so far (Deng et al. 2018), the expression of antigen in vitro becomes the first choice for vaccine preparation. For a vaccine, capsid proteins must be expressed efficiently at low production costs. The prokaryotic expression system is the most appropriate. The results of this study are conducive to the high expression of PCV3 Cap protein for the preparation of subunit vaccines to prevent and control the PCV3 infection.

In conclusion, the study proved that the main obstructive factor of the expression of PCV3 CAP protein in *E. coli* was high repetitive arginines located in its anterior region. Rare codon has few influences on its expression in *E. coli*. The truncated capsid protein without the anterior concentrated arginines could be assembled into intact viral protein. It provides a preliminary reference for the production of the recombinant PCV3 capsid protein for the vaccine or other studies.

## Data Availability

All data generated or analyzed during this study are available from the corresponding author on reasonable request.
